# Decolonizing Zemiology: Outlining and Remedying the Blindness to (Post)colonialism Within the Study of Social Harm

**DOI:** 10.1007/s10612-022-09682-5

**Published:** 2023-03-20

**Authors:** Edward J. Wright

**Affiliations:** University of Nottingham, Nottingham, UK

## Abstract

This paper hosts the first meaningful dialogue between two important epistemic movements for criminology: zemiology and decolonisation. I identify that zemiology has a disciplinary blindness to colonialism and explain this using Gurminder K. Bhambra’s scholarship—and cognate scholarship—as a frame. Three cases—Pemberton’s Harmful Societies, Grenfell, and Border Zemiology—are selected for their critical importance within zemiology. They are used to argue that zemiology works within a standard narrative of modernity characterised by capitalist nation-states, which does not recognise the colonial foundations of both of these. Capitalist modernity is, however, a colonial formation. Recognising this allows for a better understanding for a wide range of harms. I then discuss future directions for decolonial zemiology, advocating not for expansion of repertoire, but canonical revision so that colonialism is afforded space as an explanatory frame and zemiology can better explain social harm on a global level.

## Introduction

Social sciences are relatively stable in terms of their objects of study and the perspectives they offer (Szostak 2017, Becher and Trowler 2001). There are, for instance, relatively standard frames of reference for social scientists operating within particular disciplines, such as capitalist modernity for sociology (see Bhambra 2016), and the areas of study to which it exports ideas, such as criminology (Holmwood 2010). There are, however, epistemic moments in which disciplinary thresholds are ripped open and new ways of thinking about social life emerge. This paper relates to two such epistemic moments and forges a relationship between them. These are *zemiology*, which has, over approximately the past twenty years, presented a major challenge—if not an *entirely* new one (see Loader and Sparks 2011)—to criminological orthodoxy (see Canning and Tombs 2021), and *decolonization*, which has, in the twenty-first century presented a major epistemological challenge in terms of how modernity itself is to be understood (Savransky 2017). Of course, in both cases, the temporal boundaries of these movements can be broadened (Pemberton 2015; Bhambra et al. 2018), though both movements have gathered traction in the twenty-first century and each of them pose epistemological challenges to various fields of study.

Perhaps due to its recent emergence within the academy, there are discrepancies regarding how to refer to the study of harm. I use the term *zemiology* throughout this article to refer to all scholarship concerned with analysing social harm, though through doing so I know that I obscure some debate (e.g. Pemberton 2007). Furthermore, there are also some areas of critical criminology which also find ‘crime’ limiting and restricting (e.g. Cohen 1993). Whilst there is some disagreement and discussion over how this area should be best labelled, one thing is clear between these discussions: crime as a category is seen to be limiting, obscuring harms which are not criminalised, and wedding the academic study of criminology to power relations of the state.

Likewise, within the (post)colonial canon, there are some linguistic discrepancies. Bhatt (2016: 398) refers to the ‘post-/de-/colonial/-ism/-ity’ complex, indicating that various prefixes and suffixes are often interchanged within this realm. This can also be extended: Agozino (2004) has coined the term ‘counter-colonial criminology’: ‘a theory of social control from the point of view of anti-imperialist scholars who are familiar with the history of resistance to colonialist (including the colonial, postcolonial, neocolonial, internal-colonial and re-colonial) law and order reasoning’ (Agozino 2004: 350). What unites these scholars, however, is a commitment to recentre colonialism and empire within scholarship, against a knowledge base in which they have been ‘effaced from view’ (Bhambra et al. 2018: 1), to understand how the colonial informs the present, and to redress injustices that are produced through this arrangement.

Decolonization risks becoming a buzzword, one that undermines (post)colonial and indigenous struggles (see Tuck and Yang 2012). As Dhillon (2021: 255) notes, for instance, ‘The supposed progressive optics of having a more diverse student and staff body stems from a colonised ontology; that is, markers of identity (ethnicity, gender, and so on) are commodified and used to support measurable agendas that merely serve to buttress the status quo’. I do not wish to operate within such a buzzword discourse. Here, I am guided by Bhambra: in relation to their recent text *Colonialism and Social Theory*, Bhambra and Holmwood ask the question: ‘What does it mean to ‘decolonise’ a curriculum in which colonialism is not recognised?’ Their response is that ‘Paradoxically, if our book is to be understood as an attempt at ‘decolonisation’, it is one that has had to proceed by putting colonialism into the picture. In doing so we aim to create a different way of seeing sociology’. (Bhambra and Holmwood 2021: 209). Fúnez-Flores (2022: 26) has recently argued similar, noting that decolonial theory should work towards reconceptualising our current ‘social totality’ as the ‘modern/colonial capitalist world system’. It is within this remit that this paper works. It is decolonial in that it puts colonialism into a zemiological canon from which it is largely absent, and through so doing aims to create a different way of theorising harm, which locates colonial structures as a cause.

For the above, decolonisation and zemiology *should* go hand in hand. Zemiology, after all, emerged in response to criminology partly because ‘crime’ as a category ‘excludes many serious harms’ (Hillyard and Tombs 2007: 12). In the foreword to Agozino’s (2003) *Counter-colonial Criminology*, Pfohl has noted that this is a major problem for criminology:

‘there is something hauntingly unreal about a scholarly discipline dedicated to the study of crime, the criminal and the criminal law that focuses almost exclusively upon the actions of lawbreaking individuals, whilst turning a blind eye to the mass terrorism imposed upon innocent people by slavery, colonialism and their continuing legacies’.

Since this statement was made, a great deal of scholarship has emerged in this area (see, for instance, Black et al. 2021), though from the above, it is possible to say that criminology, in general, has been ignorant to the (post-)colonial. This aligns with the zemiological critique of criminology regarding the exclusion of serious harms. It also supports the zemiological critique of criminology that ‘‘crime’ serves to maintain power relations’ (Hillyard and Tombs 2007: 15). Through its narrow focus, criminology has been incapable of assessing non-criminalised harms. Those harms are non-criminalised because those who have the power to criminalise do not find it within their interests to do so. Focusing on crime as a category thereby reproduces the power dynamics that facilitated colonialism and its aftermath. Focusing on harm, however, can potentially allow knowledge that is decolonial, challenging these same power dynamics. For this, zemiology should include the postcolonial within its remit.

The decolonial potential of zemiology has not gone entirely unnoticed by criminologists concerned with colonialism. On the jacket of Canning and Tombs (2021), for instance, Agozino is highly complementary of the project. Amongst the praise that Agozino delivers, he notes that ‘the pursuit of corporate profits at the expense of human needs is the main driver of social harms’. Elsewhere though, Agozino (2018) points to the need for a recognition of imperialism as the driver of all harms, something which is, as I will argue, largely unaccounted for within the canon. As Agozino notes: ‘imperialism is the general form of all criminality’ (Agozino 2018: 356). This includes crimes of the powerful, which are not criminalised as such, but are ‘structural wrongs orchestrated institutionally’ (Agozino 2010: viii). Indeed, when Agozino writes about crime here, it could read as *harm*. On this basis, and drawing on Agozino, Choak (2020: 39–40) briefly argues similar in relation to the formation of a postcolonial feminist criminology, in which zemiological thinking could feature. There are also implications in some zemiological literature that can help locate current harmful practices as neocolonial, such as Whyte’s work (2007) on the war in and occupation of Iraq, and separately his groundbreaking work on Ecocide (Whyte 2020—discussed further below). Likewise, a special issue of *The Journal of Global Indigeneity* (see Tauri 2018a for editorial overview) centres on the promise of the lens of harm as a means of overcoming a criminological lens, which in being a colonial export and mechanism of control, cannot promote a decolonization. As Tauri (2018b: 1) notes: ‘the master’s tools will never dismantle the master’s house’, whereas indigenous understandings of and responses to harm provide the potential to promote decolonial justice.

All the above points strongly to the potential for the synthesis of decolonial theory and zemiology. As has recently been noted, however, ‘zemiology risks failing to address structural racism which is inherent to colonialities of power and thus the institutions and organisations within which we live and work’ (Canning and Tombs 2021: 108). To this extent, whilst Tombs (2018: 27) has noted that zemiology should work towards ‘post-imperial’ justice, this is largely not undertaken. As discussed further below, capitalism is identified as the structuring force of society, with the nation-state taken as a basic unit of analysis, and through this the harmful colonial foundations of the present are elided. The questions are then: how can a discipline, which is concerned with the relationship between power relations and harm ignore this issue? And how can zemiology undergo a decolonization, so that it is better equipped to address this?

## The Zemiological Critique of Capitalism and the Decolonial Critique of Social Theory

Zemiology is predominantly concerned with capitalism and its deleterious effects, and finds capitalism to provide the explanatory frame for harm. As Canning and Tombs note: Zemiology emerged as a way ‘to think anew about social harms and responses to them, increasingly produced by the profit-driven […] global capitalism’ (Canning and Tombs 2021: 23). Likewise, as Boukli and Kotzé (2018: 6) put it, those within the field have recurrently theorised that harms are ‘endured and perpetrated by those victimised by the capitalist system’. Whilst colonialism and its legacies are incredibly harmful, the colonial does not provide a frame for thinking within this field.

This style of thinking is commonplace within social science (Bhambra and Holmwood 2021). The standard social scientific narrative of modernity is one of the formations of industrial capitalist nation-states, *as if* their formation can be separated from colonialism. This has been challenged significantly by Bhambra (e.g. 2007a, 2021a, b) in collaboration with others (e.g. Bhambra and Holmwood, 2021), who has extensively revised this theorisation of modernity. The Nation-state—a key feature of (sociological narratives of) modernity—which developed during the nineteenth century, in becoming a basic unit of analysis, elides that many European states are better understood as (former) imperial polities (Bhambra 2021a), whose wealth relied upon colonial/imperial dispossession. Western industrialisation was funded through extraction of colonial wealth and resources, without which industrialisation could not have taken place, and this fuelled the formation of nation-states. The nation-state thereby becomes an enduring myth within social science (Bhambra and Holmwood 2021). From their formations onward, nation-states entailed global sociopolitical arrangements—empires—and cannot be rightly understood without recognition of this harmful underpinning. Colonialism and its aftermath are and were incredibly harmful projects, yet this does not feature within zemiological analyses, with this ‘standard’ account of capitalist modernity structuring the field.

Recently, there have been statements made towards decolonization within zemiology in recognition of a more general trend within the academy (see Canning and Tombs 2021). As this paper discusses, though, the way in which this is framed, is that zemiology might expand its repertoire, rather than fundamentally re-think its approach to theorising harm. For instance, Canning and Tombs (2021) argued for the representation of more scholars from the global south within zemiology as a means of decolonization, but do not consider what a decolonization of the existing canon would look like. The canon is one underpinned by a partial understanding of capitalism, understood in non-colonial terms. However, capitalism cannot be separated from its colonial foundations, and it is not the case that decolonization can be ‘bolted on’ to the remit of zemiology, alongside its other endeavours.

The following sections take three important examples of zemiological scholarship—Pemberton’s (2015) *Harmful Societies*, the Grenfell Tower fire, and border zemiology—and subject them to decolonial critique as per the above to suggest a re-conceptualisation of harm production in terms of a decolonial agenda. Flyvbjerg (2006: 226) notes that ‘carefully chosen’ cases are critical to the development of major scholarly theories, and accordingly the rationale for using these examples is presented below. Flyvbjerg’s (2006: 229) notion of ‘critical cases’ is of particular importance here, these having ‘strategic importance in relation to the general problem’, the general problem in this case being the lack of understanding within zemiology of colonialism and its aftermath in terms of harm production.

*Harmful Societies* is selected as it ‘remains the most developed single treatment of social harm’ (Canning and Tombs 2021: 66) and is therefore *the* key text against which to outline this problem, and against which to suggest how explanatory power over harm increases upon the adoption of a lens capable of grasping coloniality. Grenfell is selected as it is a situation over which justice is currently being sought (see, for instance, The Grenfell Tower Inquiry 2022), which has been subject to zemiological analysis (e.g. Tombs 2020). Within zemiological accounts Grenfell is theorised in relation to capitalism, ignoring a broader postcolonial discussion surrounding this. Grenfell is thus a prominent means to articulate the broader point: zemiology is yet to decolonise, but that doing so is profitable in promoting justice. Likewise, border zemiology is selected as it concerns the harms of migration—a recurrently pressing social issue on a global level (Bloch and Chimienti 2012)—on which the (post-)colonial has a bearing (Tudor 2018), and for the most part border zemiologists do not recognise. In not recognising this, harms at the border can only partly be explained, which this paper seeks to remedy. These cases are each analysed via decolonial theory, notably with Bhambra as discussed above, though with the addition of further related theorisation dependent on case.

From the above, it can be noted that this paper focuses extensively, though not entirely on the United Kingdom (UK), and to some extent Europe. This could be considered contentious, not least in that decolonization is, for some, about *re-centring* former colonies—recognising that former colonies are not merely extensions of the West, and rejecting the idea that Western consciousness and knowledge is central to all consciousness and knowledge (e.g. Mbembe 2021). This focus reflects the production of zemiological knowledge; zemiology is largely a European movement, and most scholars operating within this field are from or are based in the UK/Europe (see Canning and Tombs 2021: 42–43, and Tombs 2018). It is knowledge emanating from this European movement that is in dire need of decolonization. As Bhambra (2021b: 86) notes: ‘We are at a moment when European social theory … needs to recognise its own limitations in the face of what is necessary to overcome present inequalities and injustices’. In the UK—and Europe more broadly—colonialism as a shaping force has been obscured in social theory, and does not feature in the zemiological theorisation emanating from it. My intention is to decolonise zemiology by proceeding to put colonialism into the picture (Bhambra and Holmwood 2021) for the dominant European zemiological canon from which it is absent.

Relatedly, the canonical zemiology referred to above obscures analyses of social harm that do not specifically operate overtly under the umbrella of zemiology. This includes much scholarship produced in the Global South or within Indigenous communities that discuss and seek to undo harms, which do not work within European zemiological frames. Reconfiguring the zemiological canon so that it includes such scholarship, though it is a highly worthy cause, is a different project to what I aim to achieve here. As a reminder, following Bhambra et al. (2018) and Bhambra and Holmwood (2021) the main aim of this paper is to undo the systematic effacement of colonialism from canonical zemiology as a structure causative of harm. These projects are, however, related to one another, and I further reflect on this in the concluding discussion.

### Pemberton’s Harmful Societies

Whereas other studies typically analyse one harmful phenomenon and situate these in relation to capitalist structure to explain its causes, Pemberton (2015) undertakes a large-scale, comparative, nation-state-based analysis of harm according to various criteria and varieties of state-market relationships. For Pemberton the state has the capacity to organise aspects of capitalist societies and can mediate the harmful effects of capitalism. States can regulate markets and provide social policy frameworks as buffers against capitalist economies. States can also promote the deregulation of markets, set forth projects of privatisation, and provide minimalist social policies that do little to protect citizens against harm.

Pemberton (2015: 58–59 for full description of these types) constructs a typology, categorising nation-states according to identifiable characteristics of harm reduction. The typology is fivefold, comprising: Neoliberal, Liberal, Corporatist, Meso-corporatist, and Social Democratic. Each of these is typified by five criteria: mode of production, welfare, criminal justice, regulation, and social solidarity. Pemberton’s key finding is that all capitalist societies are harmful, though different varieties of capitalism are more/less harmful than others. Generally, those harm reduction regimes within the Social Democratic cluster (Sweden, Finland, Denmark, Norway) are less harmful than those within the Neoliberal and Liberal Clusters (UK, USA, Australia, Ireland, and New Zealand).^1^ It is the de-regulated market capitalism, the stigmatising, and means-tested assistance models of welfare designed to encourage participation in labour markets, the authoritarian approaches to justice and high imprisonment rates, that characterise these regimes and lead to these states being the worst in terms of harm reduction.

Pemberton notes that ‘the focus on capitalist harm does not seek to downplay the harms of patriarchy or neocolonialism’ (Pemberton 2015: 20). In line with the theorisation presented earlier in this article, this, however, reproduces a category error in terms of the relationship between capitalism and colonialism. To this extent, whilst Pemberton (2015: 44) notes that ‘it is undisputable that economic interdependence has increased over the last 30 years’, this is only indisputable when operating within a standard frame for understanding the development of economies on a stadial level, as per standard accounts of modernity. As Bhambra (2021a: 311) notes: ‘mercantile capitalism in the sixteenth to eighteenth centuries was not possible without the elimination and dispossession of indigenous peoples’ and ‘saw the beginnings of the systematic trade in human beings that would enslave millions of Africans’. The framing of globalisation as new—that it occurred largely over the last thirty years—and arguing that this proliferated harms primarily associated with liberal and neoliberal harm reduction regimes is mistaken once colonisation is placed in the frame (Meghji 2021). Adopting a decolonial lens, though, allows for cognition of the relationship *between* nation-states in harm production along the lines of empire. Indeed, adopting this lens disrupts the very viability of a nation-state-based analysis of harm, drawing attention to the historic interdependencies of political formations, and the imbrication of colonialism and capitalism.

That Pemberton focuses on welfare states is important to consider here, as many welfare states formed in the mid-twentieth century are indebted to colonialism (Bhambra 2021a, b). The most harmful regime type within Pemberton’s analysis—the neoliberal model—is colonial in its foundation. For instance, the ‘laggard’ (Bhambra and Holmwood 2018: 575) development of the welfare state in the USA is a matter of colonial logic; following de-segregation in the USA there followed a retrenchment of social rights. As Canning and Tombs (2021: 90) note ‘it is impossible to make sense of the harms experienced by African Americans in contemporary USA without relating these to the … legacies of colonialism and slavery’. Pemberton, however, obscures this dynamic by inheriting and using a lens deprived of the possibility of capturing the relation between colonialism and capitalism. That is, Pemberton necessarily ‘downplays’ the harms of neocolonialism. They are, however, inextricable from the form of welfare-capitalism operant in the USA.

It is also impossible to make sense of the British welfare state without relating back to those same legacies (Bhambra and Holmwood 2018). This welfare state became ‘universal’ at the point of political decolonization. The architecture of a universal welfare state was only possible in Britain through colonial wealth extraction, and, thus, from its inception onward it has always been in economic crisis. This dynamic underpins the cause of harms in Britain associated with welfare reductions. Likewise, the living conditions of those within former colonies, and the possibility of ‘harm reduction regimes’ within these polities are unextractable from the postcolonial conditions in which they are located. It is telling that Pemberton does not include any African States in his analysis, though, harms in these states, and in, for instance, Britain, are present through their relation to one another (Bhambra 2021a, b). Adopting a nation-state-based analysis, which focuses on variety of welfare-capitalism within these confines, does not capture these dynamics of harm, which a postcolonial lens would. Through a reconceptualization of harm reduction regimes according to (post)colonial relations, a different, global picture of harm emerges, one that recognises harms occurring *between* nation-states along the lines of empire.

### Grenfell Tower

The Grenfell Tower fire is understandably a focus of zemiological analysis (Tombs 2019; Canning and Tombs 2021, Cooper and Whyte 2018). On June 14th 2017, Grenfell Tower set fire, killing at least 72 people, and injuring at least a further 70 (Bulley et al. 2019). The way in which Grenfell is theorised, zemiologically, is that austere state-corporate capitalism led to poor and unsafe housing conditions, which ultimately caused its burning and the deaths and injury of those within it. A postcolonial analysis, though, provides a further means to explain this atrocity, which is unaccounted for within the canon and transcends such zemiological orthodoxy.

Beyond the immediately obvious physical harms endured by those involved in the fire—most notably loss of life and physical injury—there are other harms produced through the Grenfell Tower fire. These can be identified in relation to typologies of harm produced through zemiological scholarship (e.g. Hillyard and Tombs 2007; Pemberton 2015). In terms of *Psychological and Emotional harm* (Tombs 2019), for instance, Tombs (2019: 70) notes: ‘At least 20 survivors and witnesses of the fire had attempted to take their lives within 3 months of it’. In terms of *financial harm*, loss of property and possessions, funeral arrangements, and lost wages are some prime examples. *Cultural harm—*broadly equates to one’s capacity to participate meaningfully in public life, and to be ‘recognised’ (Yar 2012) by others of being of value. Former Grenfell Tower residents had such harm done to them: through the need for physical relocation to alternate accommodation, the possibility of participation in public life was diminished (Tombs 2019).

In a similar capacity to Pemberton’s framing, the zemiological account of Grenfell Tower is framed in terms of capitalism, as mediated by the state (Tombs 2020). As Cooper and Whyte (2018) note: it is an example of ‘institutional violence’. A retrenchment in welfare and re-articulation of government as not a buffer against inequality but championing a market which reproduces it—in short, neoliberal governance—provides a backdrop to why Grenfell happened, from this perspective. Tombs (2018, 2020) has noted similar: Grenfell is a matter of state-corporate violence under austerity. Were it not for decades of welfare retrenchment, were it not for reduction in expenditure in social housing provision, then ultimately, better accommodation would have been provided for the residents of Grenfell, built from decent materials which did not pose a threat to life.

What this explanation thus far does not provide is an understanding of why welfare cuts and why a lack of decent housing in the first place are implementable. To address these, it is necessary to engage theorisation of moral economy and its relation to monetary economy (e.g. Fraser and Honneth 2003). That is, it is necessary to think in terms of recognition, and what it means to be valued as human. Here, *cultural harm*, or rather the status of being culturally harmed, precedes, and facilitates economic harm, which produces physical harm. Indeed, as Canning and Tombs (2021: 4) have noted, former residents recognised ‘contempt as a cause of the fire … as one resident stated outside the tower as it continued to burn, “*We’re dying in there because we don’t count*.”’ (Canning and Tombs 2021: 4, emphasis added). Within zemiological accounts of Grenfell Tower, this ‘we’—beyond that it is obviously referring to residents of Grenfell Tower—is class based. It is clear from this that moral economy matters in understanding Grenfell, but whether it is a moral economy of class is only presumed in the accounts above.

Interestingly, Canning and Tombs briefly note in relation to Grenfell that ‘understanding the distributions of harms requires an understanding of north-south relations and relations of coloniality’ (Canning and Tombs 2021: 90). This operates counter to Tombs’s explanation summarised above. Although brief, it opens the possibility of a zemiological explanation of Grenfell that recognises its colonial foundation, wherein the ‘we’ of Grenfell becomes racialised in the first instance. Beyond zemiological inquiry, this has been discussed at length. To this extent, and encapsulating the debate between the zemiological analysis summarised above, and an oppositional postcolonial analysis, Danewid notes that:

‘On the night of the fire Grenfell was predominantly occupied by London’s *racialized poor*… And yet, in post-Grenfell debates about austerity, urban gentrification, and social marginalisation, race was either relatively absent or discussed in isolation from the supposedly more fundamental problem of widening class inequality under neoliberalism’ (Danewid 2020: 291, emphasis added).

Via Danewid, the ‘we’ situation becomes not one of class in isolation, but one of race. Whilst the causes of Grenfell are bound up in austerity politics, Grenfell can only be fully explained with recognition of a racialised housing market. As has been noted Tilley and Shilliam (2018: 538) in relation to Grenfell, from the 1940s ‘Black and minority ethnic residents in Britain have been relatively more likely than their white counterparts to live in ill-suited, poor-quality accommodation’. This is grounded in successive British migration policies, first produced in the early 20th century during British imperialism which reproduced racialised hierarchies of personhood, and through which ‘black and brown populations’ migrating to Britain were ‘never geographically or spatially assimilated into the wider white population’ but rather ‘excluded and sent to live 24-storeys up in towers like Grenfell’ (Perera 2020: 103). The arrangement of urban housing according to race is not merely one of class, but one underpinned by a colonial logic of personhood.

Grenfell thereby becomes just one tragic example of a broader postcolonial housing arrangement. It is not an epiphenomenon of class that ‘the majority of those who died in Grenfell Tower were people of colour, and almost a half of the residents of the tower had come to the UK from another country of birth’ (Canning and Tombs 2021: 90). To account for the harms of Grenfell, it is necessary to engage with theorisation that transcends a standard social scientific account of capitalism and engages with the postcolonial. As El-Enany (2019: 66) summarises: ‘Britain is today a space of domestic colonialism: a space of control and exclusion in which racialised populations are disproportionately subject to state violence, a violence which is uninterrogable by the law’. That this violence is uninterrogable by law provides an entry point for zemiological analysis, the notion that not all harms are criminalised being a justification for zemiological enquiry itself (Hillyard and Tombs 2007). This, however, has been largely ignored within zemiology. Whilst zemiological accounts of Grenfell transcend the official inquiry (Grenfell Tower Inquiry 2022), the lens adopted by zemiologists hinders the possibility of recognising the (post-)colonial social relations that produced it too. Adopting a postcolonial lens, though, provides a further explanation as of the physical, emotional, financial, and cultural harms endured through the Grenfell Tower fire.

### Border Zemiology

It is necessary to consider the postcolonial to understand contemporary migrations (Mains et al. 2013). Migration patterns, policies, and responses clearly have a grounding in imperial formations, which today exist as nation-states, but were brought into being as empires and colonies (Bhambra and Holmwood 2021). The harms of migration have a relation to colonialism in this way (Moreh 2021), though within border zemiology, this is not widely recognised. Recently, Moreh (2021: 429) has argued that border harms should be considered in relation to colonialism, in that ‘contemporary international migrations take place within [a] formalised nation-state system which has reconfigured colonial imaginaries of civilisation and barbarism as development and underdevelopment at a truly global scale’. Beyond this general statement on how border harms should be framed, though, this sub-area of zemiology has, surprisingly, not engaged with the postcolonial.

It is Canning (2018, 2019, 2020) whose account of harms at the border is the most developed. In summary, Canning focuses on women’s experiences of immigration detention and asylum seeking in various European contexts, most notably Britain, Denmark and Sweden. The gendered harms of such migrations, as Canning demonstrates forensically, are various: physical, economic and psychological. These harms are recognised by Canning (2020) as ‘state-corporate’ in their cause: whilst they are the result of state policies towards migrants, they are delivered by private organisations increasingly provide detention and monitoring services over such irregular migrants. Patriarchy is also considered by Canning, with this structure facilitating harms against women in these contexts.

The peculiarity of Canning’s account—given that migration studies more broadly are overtly invested in and indebted to postcolonial theorisation—is the absence of a theory of the (post)colonial, whilst analytically locating the gendered harms of migration within patriarchy and capitalism. Though Canning briefly notes that landscape of contemporary British border control is ‘neocolonial’ (Canning 2019: 41), overall, coloniality is obscured as an explanatory frame that allows for an understanding of the production of these harms. Whilst it is very necessary to account for patriarchy in understanding the gendered dimension of this harm, what is lacking here is an account of how and why immigration detention, and indeed borders more generally, exist in this harmful capacity.

Situating these systems in terms of postcolonialism allows for an explanation of how the harms caused in immigration detention are caused, and why hostile environments are in the first place produced. Canning (2019: 55) argues that ‘infantilising techniques [administered in immigration detention] are not accidental, but deliberate strategies for deterring people from staying, to instead give up on claims to asylum which might otherwise have gained refugee status’ but does not explain why it is the case that people might be deterred in the first place. The deliberate ‘degradation by design’ (Canning 2019: 46) of immigration detention is left unaccounted for: *why* is it the case that border control is deliberately designed in this way, to be degrading, controlling and violent, stripping those who are processed of ‘their autonomy and humanity’ (Canning, 2020: 262)? As per the theorisation above pertaining to Grenfell, it is necessary to engage in a discussion of the moral economy of personhood to address this question.

Elsewhere, beyond the realm of zemiology, a version of this question has been addressed: ‘the answer is in the dominance of hierarchical conceptions of human worth’ (Mayblin et al. 2020: 120), *which are rooted in colonialism, and remain characteristic of the postcolonial present.* It is pertinent that it is the language of humanity being stripped that Canning chooses to discuss the harms of immigration detention, as dehumanisation was central to the project of colonialism, and because it has been identified at length by others that immigration controls are rooted in such logic. As Mohanty (2003: 69) notes: ‘Beginning in the 1950s, British Immigration Laws were written to prevent black people (Commonwealth citizens from Africa, Asia, the Far East, Cyprus and The Caribbean) from entering Britain’. It is also therefore notable that such connections are not made in zemiological literature. Migration policy must be understood in terms of race, and this policy area must be located within the longer history of modernity, which is itself a colonial form. Following this, the harms of migration, or harms at the border, must also be understood in these terms.

Soliman (2021) moves towards such a discussion of moral economy within their zemiological account of those who die at sea whilst trying to migrate to Europe from African and Asian countries. Soliman’s (2021) entry point into discussions of harm and irregular migration is in the notion of ‘crimmigration’, which has over recent years propelled much criminological work in this area. For Soliman, however, this conceptualisation has its shortcoming in its reduction of migration control to the nation-state level. Political responses have included ‘extra-territorial migration control’—the EU’s policy of non-assistance in the Mediterranean Sea exemplifying this. To conceptualise and analyse such border harms, Soliman draws on Yar (2012: 115, emphasis in original) for whom social harm can be understood as ‘*the inter-subjective experience of being refused recognition with respect to any or all of [the following] dimensions of need*’: love, rights and esteem. Through this, harms endured by those dying at sea can be conceptualised beyond the physically immediate. That is, through Soliman’s account it is possible to recognise that: ‘The death of sea-crossers can therefore be seen not only as the immediate consequence of the lack of rescue … but are enabled by racial and financial concerns in receiving countries’ (Soliman 2021: 238).

But why are people migrating to Europe denied recognition along the lines of race in the first place? Whilst Soliman notes that ‘racial’ concerns enable such lack of action, what is not accounted for is from where ‘racial’ concerns emanate. As Tudor (2018) notes ‘the nexus of racism and migration cannot be reflected on responsibly *without taking into account Europe’s colonial past and postcolonial legacy’* (1066, emphasis added). Race evidently matters here, as Soliman suggests, though without an account of the structure productive of race, the aetiology of border harms remains absent. The Mediterranean Sea is not only ‘the EU’s southern border’ (Soliman 2021: 232) but the Northern Border of Africa, and this has ‘postcolonial implications’, emblemising that contemporary irregular migration patterns follow colonial fault lines (Franko 2021: 393–394). Indeed, Franko conceptualises Frontex—the European Border and Coast Guard Agency—in terms of conquering and domination, and elsewhere decolonization movements have campaigned against its operations (Decolonizing Development 2020). Whilst Soliman’s zemiological account might be preferable in that it overcomes the lens of crimmigration, which ‘obscures the global dimensions of migration control’ (Soliman 2021: 232), it also obscures *the coloniality* of the global dimensions of migration control, and how this facilitates a lack of recognition.

In summary, colonial relations explain contemporary border harms, though this is unaccounted for within major zemiological accounts. If a zemiological approach is going to be proffered as a preferable means of making sense of migration control in a global world (Soliman 2021; Canning 2018; Canning and Tombs 2021), then it must further engage with a theorisation of modernity as (post)colonial. Considering the colonial foundations of border harms is a necessary development within border zemiology, as is considering the colonial foundations of modernity—and harm—for zemiology more broadly.

## Future Directions for Decolonial Zemiology

Between the above examples, there is a recurrent theme, that the (post)colonial is elided within zemiology. In all cases, however, engaging with such scholarship provides further explanation of various harms between different contexts, and works towards a fruitful decolonization within the field. With the above as precedent, this section engages with Canning and Tombs’s (2021) statements on future directions for zemiology, which call for a greater emphasis on decolonization within the field. As I will argue, whilst this call is welcome, it is ultimately insufficient, as it allows for the core research programme for zemiology to remain intact, and not subjected to its own decolonization. Accordingly, the invitation for zemiology to address its core future research programme is offered.

Canning and Tombs (2021) provide an outline for a future research agenda for zemiology, including an emphasis on ‘neocolonial harms’ (Canning and Tombs 2021: 116), with the following provided as examples: *trade in conflict minerals*, *tourism*, *racism* and *global inequalities*. Alongside but separate to these neocolonial harms (see Canning and Tombs 2021: 116) listed as categories for investigation are, for instance: *harms and border controls*, *green harms* and the *harms of legal drugs*. Harms and border controls contain the following subthemes: The refugee ‘crisis’; Points-based immigration systems; ‘crimmigration’; far-right groups. The harms of legal drugs contain the following subthemes: tobacco; alcohol; the transatlantic trade in sugar; pharmaceuticals and pharmaharms. All these harms are positioned within Canning and Tombs’s agenda as separate to ‘neocolonial’ harms, though as this paper argues, many harms, not currently understood as having a bearing to colonialism, *should* be understood in this way. As already discussed, for instance, border harms are underpinned by a colonial logic. Similarly, far-right groups tend to be formed around ‘white’ national identities and cannot be fully understood without reference to colonialism. Positing that future directions for zemiology could include neocolonial harms *alongside* a plethora of other harms is, therefore, mistaken.

In relation to green harms, for instance, to articulate this point, it is possible to draw upon Whyte’s analysis of ecocide.^2^ Though Whyte does not closely frame his analysis (Whyte 2020) in terms of zemiology and harm, he is more broadly associated with zemiology as a movement, and his work does have a strong bearing on environmental harms (see also Crook et al. 2018). Ecocide, after all, pertains to the ‘deliberate destruction of our natural environment’ (Whyte 2020: 2). Whyte focuses on the ecocidal corporate practice of Stora Enso, a Northern European company, with roots in Germany and Switzerland, though now based in Helsinki, Finland. Stora Enso is ‘one of Uruguay’s biggest land owners’ (Whyte 2020: 66) and also owns a major plantation in Brazil. Stora Enso is routinely criticised by Uruguayans for destruction of land, and through this the destruction of any sovereign future. Against this, Stora Enso claims an ethos of sustainability. As Whyte (2020: 68) notes: ‘The claims transnational corporations make as the guarantors of environmental sustainability contain precisely the same hypocrisy that we find in the classic colonisers’ claim to be civilising the savages’. For all of this, Stora Enso is located by Whyte as a neocolonial corporation, which retains a logic of colonial expansion, through a primacy given to economic productivity over way of life. Even a cursory reading of Whyte’s work therefore allows for recognition that locating neocolonial harms as an area for investigation, alongside but separate to green harms, is mistaken. As Whyte (2020: 69) notes: ‘colonial capitalism was always ecocidal’, and contemporary transnational corporations reproduce this logic. Future green zemiological projects should develop on Whyte’s analysis, and in doing so further develop theorisation which recognises that green harms are neocolonial harms, underpinned by colonial logics renewed through capitalist conquest in the twenty-first century.

A similar argument can be made in relation to legal drugs. Tobacco is a legal substance that is harmful, and should form part of the research agenda for zemiology, but *within* rather than *separate to* its decolonial agenda. Whilst tobacco was produced on local, subsistence levels prior to colonialism (Goodman 1993) it is only with its European ‘discovery’ that it became a globally consumed product, and this is only the case due to colonial extraction. For instance, the history of Malawi is one of colonialism, through its tobacco production. In 1891, Malawi became a protectorate of the United Kingdom, and with this colonisation tobacco production increased by about 75% annually over the next twenty years. In the early twentieth century, as part of this arrangement, *British American Tobacco* built a factory in Malawi to process tobacco for the European market, and it became the largest supplier of tobacco in the world outside of the USA. Malawi became politically independent in 1970, though the presence of *British American Tobacco* remained, and today, there is a system of contract farming ‘reminiscent of the colonial system’ (Smith and Lee 2018: 197). In other words, the tobacco industry is a harmful, *neocolonial arrangement*, which has come into being through its formal colonial history and can be understood as an extension of it. Underpinning the physical harms of smoking is a colonial arrangement, which has entailed financial harm to entire countries, and has kept impoverished those farmers who grow and produce tobacco, which is facilitated by a racialised articulation of personhood and moral value.

‘Pharmaharms’ can be also understood through a colonial lens. Historically, the development of pharmaceuticals is bound up in relations between colonial administrations and private industry, in that the latter sought to develop therapies for ‘tropical’ diseases (Baxerres and Cassier 2022: 2). More recently, of course, the COVID pandemic has in various ways affected the global south, crucially here, in terms of vaccine distribution on a global level. As noted by Hassan et al. (2021: 1) the ‘lack of vaccines in the global South means that the health hospital admissions, and deaths will fall disproportionately on these countries’. This is locatable in what Hassan et al. (2021) refer to as paternalism and racism, which feature more broadly in the global political economy of medicine. It is of course not only COVID 19 which becomes understandable in these terms. The general category of ‘aid’—including pharmaceutical aid—who sends and who receives it, maps onto colonial fault lines.

Overall, then, positing that future directions for zemiology could include neocolonial harms *alongside* a plethora of other harms is too limited. Expansion of remit without a reconfiguration of thought does not equate to decolonization. Colonial relations are a *cause* of harms, rather than another area of content for investigation. A zemiology which takes decolonization seriously cannot only consider the colonial amongst an expanding repertoire of subjects. Rather, it must reconsider how colonialism *produces* harms across explananda. Taking the (post)colonial as an explanation for social harm has largely been ignored within this area of study, which works within a standard social scientific account of modernity. This takes the nation-state and capitalism as the basic frames for analysis, ignorant to the colonial foundations of both. Recognising that the foundations of the present are colonial, however, and that colonial relations are variously renewed in the twenty-first century provides a greater understanding of harm, currently unaccounted for in this area of study.

## Discussion and Conclusion

There are some efforts (e.g. Agozino 2018; Tauri 2018a, 2018b; Choak 2020; Canning and Tombs 2021) that point towards the possibility of and desirability for a decolonial zemiology. There has, however, been little attempt to systematically and purposefully decolonize zemiology. Nor is there a meaningful and elaborated recognition within the Eurocentric zemiological canon of colonialism and its aftermath as causative of harm, when this is so obviously the case. This is surprising, given that zemiology has always been concerned with power relations, harms perpetrated by states, and those facets of life that are harmful though not criminalised. Zemiology works largely within a standard social scientific frame, which takes the capitalist nation-state as its frame for analysis, which elides the colonial formation of modernity, and with this the possibility of conceptualising harm in these terms. Adopting a lens which transcends this standard social scientific framing of modernity, and recognising that modernity is a colonial formation, however, opens zemiology to further theorisation of a plethora of harms.

In addressing an entire area of study, this article has necessarily had to work with specific examples. The first of these, which is discussed as a landmark study within this field (Canning and Tombs 2021), is Pemberton’s *Harmful Societies* (2015). Whilst supremely ambitious in scope, adopting a postcolonial lens for this study would have produced a different global topology of harm. Moreover, whilst Pemberton argues that focusing on the harms of different welfare capitalist formations does not seek to ignore the harms of colonialism, this very statement contains a category error. Welfare capitalist formations are colonial formations. Likewise, by focusing on harm reduction regimes at the national level, harms *between* nations on a global level are obscured. The harms in contemporary Britain, though, formed through a neoliberal harm regime are necessarily indebted to colonial economies. Similarly, the harms endured in former colonies are related to their colonial past.

In the case of Grenfell, it was identified that the zemiological aetiology of the fire was neoliberal capitalism. However, as Canning and Tombs have recently noted, the majority of people affected by this fire were not white, and, as this paper argues, this is not coincidental, nor an epiphenomenon of neoliberal capitalism. Rather, Grenfell was a racialised form, indebted to a postcolonial geography of the city, premised upon a moral economy of racialised personhood. Such discussion cannot be ignored in explaining Grenfell, though, within major zemiological accounts this has been elided.

The third case considered was border zemiology. Nation-state borders as they exist today are a legacy of colonialism, and the policing and protection of borders reproduces coloniality. This is thoroughly rehearsed within migration studies, though zemiological analyses are all but blind to their formation in this way. That harms are administered at European borders is a matter of racial hierarchy, one produced through a colonial conceptualisation of the subject. For a discipline wishing to address harms at the border, it is entirely necessary to engage with such theorisation. This paper provides an entry point to do so, by considering how Yar’s framework of recognition can be applied to contemporary migration issues, whilst recognising the colonial undergirding to harms in this context.

Though there have been recent calls for zemiology to decolonize, this call has been made in such a way that leaves the kernel of the research programme intact. This should not remain the case. There have been endeavours in other disciplines to address such core ideas in a decolonial way, which have renewed analysis and promoted the development of new knowledge. Within sociology, for instance, modernity—its foundational area of enquiry—has been excavated (e.g. Bhambra and Holmwood 2021), to suggest that its roots are in colonialism, which challenges a whole host of categories and concepts and their relations to one another (e.g. capitalism, colonialism, class, race, the nation-state, migration). In a bid to begin a discussion which disrupts the core of zemiology in a similar way, this article has suggested ways in which future zemiological scholarship might reconfigure what can be considered through a postcolonial lens. Many harms for investigation situated by Canning and Tombs outside of a neocolonial agenda are matters in which the (post)colonial becomes explanatory. Diverse areas for investigation—far-right groups, the green harms of ecocidal corporations, tobacco production, and consumption—were here drawn on, and in each case, analysis indicated that postcolonial relations become important to consider in explaining harm production. Though this article has necessarily worked with specific examples, a more general point can be derived: zemiology is lacking an understanding of the postcolonial condition, and of decolonization, and should seek to revise its canon towards decolonial ends. Doing so furthers analysis of many harms endured in contemporary societies, and cannot be ignored, as it has been, within this area of study.

Through a meaningful decolonization, zemiology would be armed with means to recognise harms beyond those caused by capitalism, to meaningfully work towards post-imperial justice, as is Tombs’s (2018) unrealised suggestion. It would also place—as this article has done—zemiology into dialogue with wider decolonial discussions from other disciplines: sociology, criminology, politics, international relations, history, and geography, for instance, which would promote a wider decolonisation. Through such dialogue, it might be that zemiological theorisations could become part of a decolonial toolkit to analyse and address the harms of (post-)colonialism, given that zemiology has the means to dissect harm as a category. Further decolonial work—how should harm be conceptualised, so that it can identify and address the harms of postcolonialism?—should be undertaken here.

Relatedly, the purpose of this paper was not to work towards redefining harm or expanding its conceptual remit through a redrawing of canonical lines to include scholarship from the Global South which theorises harm. This, however, does deserve some comment here. Cunneen and Tauri (2019: 377) note that ‘there needs to be a willingness on the part of criminology to learn from Indigenous peoples who have their own ways of knowing and responding to crime and social harm’. The ways in which harm is defined and conceptualised within zemiology is distinctly Western (see Canning and Tombs 2021; see also Bhambra 2007b, on the division between system and social in European sociology). Following Cunneen and Tauri, there does indeed need to be a willingness within zemiology to learn about such ways of knowing and responding to social harm.

One initial example here is *Gacaca*—a form of justice in Rwanda, which focuses reconciliation and social harmony, and focuses on collective responsibility for harm, rather than being limited to a focus on the individual (Tamale 2020). This form of justice was re-introduced in Rwanda after its genocide, which has its roots in Belgian colonisation (Tamale 2020) as the ‘colonial justice system was ill-equipped to deal with the enormity of the tragedy’ (Tamale 2020: 157). Acknowledging social formulations surrounding harm, such as Gacaca, would work towards decolonizing zemiology in a different though complementary way to this paper. It would respond to the ‘intellectual violence of colonialism’: ‘the discrimination and denial of any alternative ways of thinking, knowing, and being in the world’ (Dimou 2021: 432). Indeed, the *epistemic harm* of colonialism involves the imposition of a ways of life—for instance, schemas of thought, perception, action, time—by settler-colonists (Tamale 2020). Canonising the harms to ways of life instilled upon those communities would work to open further zemiology to the harms of colonialism. This would work towards cognitive justice (de Sousa Santos 2017) and addressing the epistemic harm of colonialism.

As with many critical endeavours, zemiologists (e.g. Tombs 2018) have been guided by Marx, eager to point out that the study of harm should not remain an armchair exercise. *The philosophers have only interpreted the world in various ways; but what matters is to change it.* The very way in which thinking and acting are posited as oppositional, or separate categories, however, is problematic. *Thinking is action*, and this paper contributes to changing the world through providing a new lens through which harm should be addressed, one that responds to calls from within zemiology (Canning and Tombs 2021) to consider colonialism and its aftermath further. Nigam has similarly noted, in relation to Marx’s eleventh thesis and decolonization, that:

‘The point is to change the way we do theory … the eleventh thesis was not just a crude call to do away with theory and focus on the ‘real task’ of changing the world, although it has been unfortunately interpreted in that fashion by generations of Marxists (Nigam 2020: xiii).

Theory is something that is done, and it can and does change the world, in terms of how others see the world and act in it. Decolonization is an ongoing process (Gopal 2021), and what this paper represents is a significant movement towards thinking in a decolonial way about harm, so that a plethora of harms can be better understood. The topics discussed in this paper should not be understood to represent a decolonial zemiological agenda in its entirety, rather they are examples of through which I aim to promote the action of thinking about harm in a decolonial way, and to suggest that a decolonial lens should be taken up by those working within zemiology to fruitful ends. Only through re-orientation towards a theoretical account of postcolonial social relations will harms be fully explained, and in time, undone.

## Footnotes

1 Across different criteria these states are placed in these two types.

2 Interestingly, ecocide does feature in Canning and Tombs’s (2021) outline of future directions for zemiology, but under the heading of harms and armed conflict, rather than green harms. This placement is confusing, in that most immediately, Whyte (2020) locates ecocide as a ‘green’ issue, and does not locate ecocide in terms of ‘armed conflict’ to the same extent.
